# The first-in-class alkylating deacetylase inhibitor molecule tinostamustine shows antitumor effects and is synergistic with radiotherapy in preclinical models of glioblastoma

**DOI:** 10.1186/s13045-018-0576-6

**Published:** 2018-02-27

**Authors:** Claudio Festuccia, Andrea Mancini, Alessandro Colapietro, Giovanni Luca Gravina, Flora Vitale, Francesco Marampon, Simona Delle Monache, Simona Pompili, Loredana Cristiano, Antonella Vetuschi, Vincenzo Tombolini, Yi Chen, Thomas Mehrling

**Affiliations:** 1 Laboratory of Radiobiology, Department of Applied Clinical Sciences and Biotechnologies, University of L’Aquila, L’Aquila, Italy; 2 Division of Radiotherapy, Department of Applied Clinical Sciences and Biotechnologies, University of L’Aquila, L’Aquila, Italy; 3 Division of Neurosciences, Department of Applied Clinical Sciences and Biotechnologies, University of L’Aquila, L’Aquila, Italy; 4 Division of Applied Biology, Department of Applied Clinical Sciences and Biotechnologies, University of L’Aquila, L’Aquila, Italy; 5 Division of Human Anatomy, Department of Applied Clinical Sciences and Biotechnologies, University of L’Aquila, L’Aquila, Italy; 6 Laboratory of Applied Biology, Department of Life, Health and Environmental Sciences, University of L’Aquila, L’Aquila, Italy; 7 Division of Radiotherapy, Department of Experimental Medicine, University of Rome “La Sapienza”, Rome, Italy; 8 Northlake International LLC, Pleasanton, CA USA; 9 Mundipharma-EDO GmbH, Basel, Switzerland

**Keywords:** Glioblastoma, Bendamustine, Vorinostat, Tinostamustine, Radio sensitivity

## Abstract

**Background:**

The use of alkylating agents such as temozolomide in association with radiotherapy (RT) is the therapeutic standard of glioblastoma (GBM). This regimen modestly prolongs overall survival, also if, in light of the still dismal prognosis, further improvements are desperately needed, especially in the patients with O6-methylguanine-DNA-methyltransferase (MGMT) unmethylated tumors, in which the benefit of standard treatment is less. Tinostamustine (EDO-S101) is a first-in-class alkylating deacetylase inhibitor (AK-DACi) molecule that fuses the DNA damaging effect of bendamustine with the fully functional pan-histone deacetylase (HDAC) inhibitor, vorinostat, in a completely new chemical entity.

**Methods:**

Tinostamustine has been tested in models of GBM by using 13 GBM cell lines and seven patient-derived GBM proliferating/stem cell lines in vitro. U87MG and U251MG (MGMT negative), as well as T98G (MGMT positive), were subcutaneously injected in nude mice, whereas luciferase positive U251MG cells and patient-derived GBM stem cell line (CSCs-5) were evaluated the orthotopic intra-brain in vivo experiments.

**Results:**

We demonstrated that tinostamustine possesses stronger antiproliferative and pro-apoptotic effects than those observed for vorinostat and bendamustine alone and similar to their combination and irrespective of MGMT expression. In addition, we observed a stronger radio-sensitization of single treatment and temozolomide used as control due to reduced expression and increased time of disappearance of γH2AX indicative of reduced signal and DNA repair. This was associated with higher caspase-3 activation and reduction of RT-mediated autophagy. In vivo, tinostamustine increased time-to-progression (TTP) and this was additive/synergistic to RT. Tinostamustine had significant therapeutic activity with suppression of tumor growth and prolongation of DFS (disease-free survival) and OS (overall survival) in orthotopic intra-brain models that was superior to bendamustine, RT and temozolomide and showing stronger radio sensitivity.

**Conclusions:**

Our data suggest that tinostamustine deserves further investigation in patients with glioblastoma.

**Electronic supplementary material:**

The online version of this article (10.1186/s13045-018-0576-6) contains supplementary material, which is available to authorized users.

## Background

High-grade gliomas are the most frequent and aggressive primary brain tumor in the adults. Grade IV gliomas named also glioblastoma (GBM) show a devastating clinical evolution, a short survival, and a poor quality of life. Currently, therapeutic standards show 27 and 10% of survival when considered the analyses at 2 and 5 years from diagnosis [1]. Although the benefits are modest and of limited duration, temozolomide-based chemotherapy is part of the standard treatment for GBM in the first-line and recurrent settings [2]. Temozolomide is an alkylating agent that triggers DNA damage [3]. Molecular pathways (direct DNA damage reversal, base excision repair [BER], and mismatch repair [MMR]) are activated after alkylation DNA damage as mechanisms of defense towards cell death or large mutations. Temozolomide (TMZ) is a second-generation imidazotetrazine lipophilic prodrug that crosses the blood-brain barrier (BBB) and induces GBM cell death by introducing alkyl groups into DNA cross links [3]. This drug forms O6-methylguanine, N3 methyl adenine, and N7 methylguanine adducts leading to the formation of single- and double-strand DNA breaks associated with both apoptosis and senescence of tumor cells [4]. Temozolomide resistance involves, indeed, both O6-methylguanine-DNA-methyltransferase (MGMT)-dependent and MGMT-independent mechanisms. MGMT protects the cellular genome from the mutagenic effects of TMZ by removing the O6-alkylguanine DNA adducts [5], thereby reducing the effect of TMZ. MGMT promoter methylation status is responsible for regulating MGMT expression and has been statistically significantly correlated with increased survival in GBM patients receiving the standard treatment (49 versus 15%, 2-year survival rate) [5, 6]. The MMR system is critical for the maintenance of replication fidelity and for inducing appropriate cellular responses to DNA damage [4]. In mismatch repair, MMR-deficient cells may become tolerant to the mispairing of O6-methylguanine with thymine. N3 methyl adenine and N7 methylguanine may be repaired by BER enzymes including MPG, 3-methylpurine-DNA glycosylase. Maximal safe surgical resection followed by RT with concomitant TMZ administration indicates that patients have a post-operative expected survival of 12–15 months [1, 6]. Tumor recurrence is mediated by the recruitment of glioma stem-like cells named also as tumor-initiating cells (TIC). This event is often associated also with resistance to standard therapies [7, 8].

Bendamustine (BDM) is an alkylating chemotherapeutic agent displaying a unique pattern of cytotoxicity compared with TMZ [9]. This is a bifunctional mechlorethamine derivative with properties similar to those seen with cyclophosphamide, chlorambucil, and melphalan, containing a purine-like benzimidazole ring similar to cladribine. This molecule was designed to have both alkylating and anti-metabolite properties. Differences with other alkylating agents have been observed in regard to its effects on DNA repair and cell cycle progression. Moreover, BDM can induce cell death through both apoptotic and non-apoptotic pathways, thereby retaining activity even in cells without a functional apoptotic pathway. BDN appears to have only a partial cross resistance to other alkylating agents [9–11] and was used as a salvage therapy monotherapy for recurrent GBM [12]. Histone deacetylases (HDAC) are frequently overexpressed in tumors including GBMs [13] and control the gene expression, cell proliferation, and drug resistance of tumor cells [14]. Recently, we observed that high-intensity HDAC4 and/or HDAC6 immunostaining was predictive of poor clinical outcome in patients with GBM treated with standard chemo-radiotherapy [13]. In vitro experiments demonstrated also that silencing of HDAC4 or HDAC6 was able to radio-sensitize resistant U87MG and U251MG GBM cell lines through the promotion of DNA double-strand break (DSB) accumulation and down-modulation of DSB repair molecular machinery activity. HDAC inhibitors (HDACi) represent anti-cancer drugs that alter both the epigenome (and this is the gene expression) and the function of crucial non-histone protein. It has been widely demonstrated that administration of HDACi modulates the differentiation status [14–17] of glioma cells through modification of expression levels of differentiation or stemness proteins [14] as for example GFAP, β-catenin, or nestin. The detection of these proteins together to stem cell markers (CD133, CD44, and Sox2) may help to discriminate glioma cells to glioma stem-like cells [16, 17]. They also exert a synergistic effect with, or additive to, other anticancer treatments, including RT [18, 19]. While vorinostat (suberoylanilide hydroxamic acid, SAHA) and panobinostat have undergone equal evaluation as combination therapies for TMZ in glioblastoma [20, 21], of the potential for HDACi may be as a radio-sensitizer inducing chromatin relaxation, altering transcription of DNA damage repair genes and common cell death pathway synergisms [22]. Furthermore, despite increasing the efficacy of chemotherapeutic agents such as TMZ [22, 23], HDAC inhibitors may potentiate the evolution of acquired TMZ resistance linked to MGMT upregulation in glioblastoma xenografts [24]. Tinostamustine (EDO-S101; TINO) is the first-in-class alkylating deacetylase inhibitor (AK-DACi) molecule which was designed to create a very potent cytotoxic agent for systemic use, with the aim of increasing the efficacy of the alkylating DNA damage through deacetylase-mediated chromatin relaxation. In TINO, the active structures of SAHA and BDM were fused together to create a completely new chemical entity. Preclinical experiments and biochemical characteristics are shown in references [25–27] and demonstrated that TINO exerts significant activity against several hematological malignancies. In addition, the molecule is active in primary resistant cells as well as in cells that have acquired resistance [26].

The aim of this study is to investigate the antitumor effects of TINO in association with radiotherapy in preclinical models and to compare activity with standard of care.

## Methods

### Reagents and drugs preparation

Plasticware and materials for tissue culture were purchased from Euroclone (EuroClone S.p.A, Milan, Italy). Antibodies for β-actin [sc-130065], acetylated-histone H3 (Lys 24) [sc-34262], and total histone H3 (Ser 28) [sc-24516], GFAP (2E1) [sc-33673], nestin [sc-23927], β-catenin [sc-7199], LC3-II [sc-271625], beclin1 [sc-48341], and p62 [sc-28359] were purchased from Santa Cruz (Santa Cruz, CA, USA). Survivin antibody was purchased from Biorbyt (Cambridge, UK). Antibodies against E-cadherin and MIB1 (Ki67) were purchased from Dako (Carpenteria, CA). We used the ApopTag® peroxidase in situ apoptosis detection kit purchased from Merck Millipore (Merck, Milan, Italy). Vessel count was detected by using anti-mouse CD34 from eBioscience, Inc. (Prodotti Gianni SpA, Milan Italy). Bendamustine HCl, TMZ, and vorinostat (SAHA) were purchased from Selleckchem (Aurogene, Rome Italy). Tinostamustine (TINO) was provided by Mundipharma-EDO GmbH. For in vitro cell viability assays, TINO was dissolved in dimethyl sulfoxide (DMSO) and used at final concentrations of < 0.5% DMSO. For in vivo test, TINO was prepared as an intravenous injection solution containing 15% HPBCD, 1.5% acetic acid, and 1.25% NaHCO3. Since TINO undergoes hydrolysis in aqueous solution, it is very important to immediately dose the animals after each fresh formulation is made.

AT101, a small Bcl2 inhibition molecule, was kindly provided from Jeffrey M. Brill (Ascenta Therapeutics, Inc., Malvern, PA). AT101 was dissolved in DMSO to obtain a stock solution of 100 mM. and used at 5 μM in culture.

### Cell lines

Twelve human glioma cell lines (U251MG, U373, U118, U138, A172, U87MG, LN19, SW1783, SNB19, LN229, T98G, and D54) were cultured at 37 °C in 5% CO2 and were maintained in Dulbecco’s modified Eagle’s medium (DMEM) containing 10% (*v*/*v*) fetal bovine serum, 4 mM glutamine, 100 IU/ml penicillin, 100 μg/ml streptomycin, and 1% nonessential amino acid (Thermo Fisher/Life Technologies, Inc., Carlsbad, CA, USA). To minimize the risk of working with misidentified and/or contaminated cell lines, we stocked the cells used in this report at very low passages and used < 20 subcultures. Periodically, a DNA profiling by GenePrint® 10 System (Promega Corporation, Madison, WI) was carried out to authenticate cell cultures. Karyotypically distinct U251MG, SNB19, and U373 cell lines were defined to be the same origin but have different drug treatment sensitivities. Similarly, U138-MG cells show strong similarity to U118-MG cells, sharing at least six derivative marker chromosomes. Luciferase-transfected U87MG cells were kindly provided by Jari E. Heikkila, Department of Biochemistry and Pharmacy, Abo Akademi University, Turku, Finland. Six GBM patient-derived stem cell lines (BT12M, BT25M, BT48EF, BT50EF, BT53M), kindly provided by J. Gregory Cairncross and Samuel Weiss (Hotchkiss Brain Institute, Faculty of Medicine, University of Calgary, Calgary, Alberta, Canada) [28] and GSCs-5 and CSCs-7 [29] from Marta Izquierdo (Departamento de Biología Molecular, Universidad Autónoma de Madrid, Spain) were maintained as neurosphere cultures in Neurocult medium (Stem Cell Technologies, Vancouver, BC, Canada) supplemented with epidermal growth factor (20 ng/ml) and fibroblast growth factor (10 ng/ml). GSCs-5 cells were transfected with pGL4.13 vector (Promega Italia, Milan) using jetPEI (Polyplus) to create a stable luciferase expression clone selected by limited dilution.

### Cell viability assay

The cytotoxicity of TINO, BDM, and TMZ was measured by the Cell Counting Kit-8 (CCK-8; Dojindo Molecular Technologies Inc., Tokyo, Japan) as suggested by its producer. The optical density (OD) values were averaged and normalized against the controls to generate dose-response curves to calculate the IC50 values using Grafit software.

### Cell cycle and apoptosis analysis

SubG1 cell percentage (resulting in a mixture of necrosis, autophagy, and apoptosis) was detected with Propidium Iodide (PI) by using Tali™ instrument (Thermo Fisher Scientifics, Carlsbad, CA, USA). Apoptosis was analyzed using Alexa Fluor® 488 Annexin V/Dead Cell Apoptosis Kit (Life Technologies Europe BV, Monza, Italy). All cells were then measured on a Tali® Image-Based Cytometer measuring the fluorescence emission at 530 nm (e.g., FL1) and > 575 nm. The results were expressed as the percentage of cell death by apoptosis in controls and in treated cultures.

### HDAC activity assay

HDAC activity was evaluated by a colorimetric HDAC activity assay kit (Enzo Life Sciences GmbH) in nuclear extracts of cells treated as previously described [30].

### Clonogenic survival assay

For clonogenic survival, exponentially growing cells (70% confluence) were cultured in regular media and treated with TINO, at the appropriate concentrations, or vehicle (final DMSO concentration of 0.1%) for 24 h. Tumor cells were then irradiated at room temperature with increasing doses of radiation (0–6 Gy) using an X-ray linear accelerator (dose rate of 200 cGy/min). Non-irradiated controls were handled identically to the irradiated cells, with the exception of the radiation exposure. After treatment, cells were diluted at the appropriate concentration (1000 cells) and re-seeded into a new 100-mm tissue culture dish (in triplicate) and incubated for 14 days. At day 14, the media was removed and colonies were fixed with methanol:acetic acid (10:1, *v*/*v*) and stained with crystal violet. Colonies containing > 50 cells were counted. The plating efficiency (PE) was calculated as the number of colonies observed/the number of cells plated; the surviving fraction (SF) was calculated as follows: colonies counted/cells seeded X (PE/100). The mean inactivation dose was calculated according to the method of Fertil [31], and the cell survival enhancement ratio (ER) was calculated as the ratio of the mean inactivation dose under controlled conditions, divided by the mean inactivation dose after drug exposure, as described by Morgan [32]. We also used the “dose enhancement factor” as the ratio between “dose with radiation alone and dose with radiation + drug”. A value significantly > 1 indicates radio-sensitization.

### Comet assay

The comet assay was carried out by using the OxiSelect™ 96-well Comet Assay Kit (Cell Biolabs Inc., San Diego CA) and measured as suggested by its producer. Analyses were performed in triplicate and presented data represent mean ± standard error (SE) and consider three replicated experiments.

### AVO staining

AVO staining was used to detect the presence of acidic vesicles after treatments. Cells were treated with a final concentration of 1 μM of acridine orange solution at 37 °C for 15 min, washed in phosphate-buffered saline (PBS), and observed under a fluorescence microscope (excitation = 488 nm, emission = 520 nm) (Carl Zeiss, Germany).

### Western blotting

Following treatments, cells, grown in 90 mm diameter Petri dishes, were washed with cold PBS and immediately lysed with 1 ml lysis buffer containing a proteinase and phosphatase inhibitor cocktail. Total lysates were electrophoresed in 7% SDS-PAGE, and separated proteins were transferred to nitrocellulose and probed with the appropriate antibodies using the conditions recommended by the suppliers. Total extracts were normalized by using an anti-β-actin antibody.

### ELISA determinations

After appropriate treatments, floating and adherent cells (obtained from the medium and a PBS wash, or after trypsinization, respectively) were pooled and pelleted by centrifugation. Cell pellets were washed with PBS, lysed with RIPA buffer, and assayed by ELISA determinations for (i) active human Caspase-3 (CBA045, Merck Chemicals Ltd. Nottingham, UK), Beclin-1 protein expression (E98557Hu, USCN life sciences, Houston, TX, USA) and DNA damage assay (EpiQuik in situ DNA Damage Assay Kit; Epigentek, Farmingdale, NY, USA). All experiments were performed following the manufacturer’s protocols. Analyses were performed in triplicate and presented data represent mean ± standard error (SE) and consider three replicated experiments. APOSTRAND™ ELISA apoptosis detection kit (code BML-AK120-0001) and p62 ELISA kit (code ADI-900-212-0001) were purchased from Enzo Life Sciences, Inc. (Farmingdale, NY, USA). Beclin 1 ELISA (code SEJ557Hu) was purchased from 2BScientific (Heyford Park, UK). Human MAP1LC3B / LC3B ELISA Kit was purchased from LifeSpan BioSciences (Seattle, WA, USA).

### Mouse glioblastoma xenograft model

Female CD1-nu/nu mice, at 6 weeks of age, were purchased from Charles River (Milan, Italy) under the guidelines established by our Institution (University of L’Aquila, Medical School and Science and Technology School Board Regulations, complying with the Italian government regulation no. 116 January 27, 1992 for the use of laboratory animals). All mice received subcutaneous flank injections (two each) of 1 × 10^6^ U251, U87MG and T98G cells representing models for MGMT negative, MGMT gene methylated and MGMT positive cells. Tumor growth was assessed bi-weekly by measuring tumor diameters with a Vernier caliper. If we considered a xenograft as equivalent to an ovoid having three diameters: the formula used was ‘TW (mg) = tumor volume (mm^3^) = 4/3πR1 × R2 × R3 in which R1/R2/R3 are the 1/2 diameters (rays), shorter diameter is the thickness/height of tumor, larger diameters are the length and width of tumor [33–35]. At about 10 days after the tumor injection, 30 mice with tumor volumes of 0.5~ 0.8 cm^3^ were retained and randomly divided into six groups (five mice per group with two tumors each) named (1) control (vehicle), (2) TINO (60 mg/kg at days 1, 8, and 15 q28 days, iv), (3) radiotherapy (RT, 4 Gy delivered in a single fraction [34]), and (4) TINO plus RT. These treatments were compared with standard therapies consisting of TMZ and TMZ plus RT. Therefore, two additional groups were included: (5) TMZ (16 mg/kg/5 consecutive days) and (6) TMZ plus RT. Anesthetized tumor-bearing mice received a focal irradiation. All mice were shielded with a specially designed lead apparatus to allow irradiation to the right hind limb. Mice were kept under these conditions until all irradiation finished. At the end of experiments (35 days after the start of treatments), animals were sacrificed by carbon dioxide inhalation and tumors were subsequently removed surgically. Half of the tumor was directly frozen in liquid nitrogen for protein analysis and the other half fixed in paraformaldehyde overnight for immunohistochemical analyses. Indirect immunoperoxidase staining of tumor xenograft samples was performed on paraffin-embedded tissue sections (4 μm). Tumor microvessels were counted at × 400 in five arbitrarily selected fields, and the data were presented as number of CD34^+^ mouse microvessels/× 100 microscopic field for each group. Ki67 labeling index was determined by counting 500 cells at 100× and determining the percentage of cells staining positively for Ki67. Apoptosis was measured as the percentage of tunnel positive cells ±standard deviation (SD) measured on five random fields (400×).

### Evaluation of treatment response in vivo (xenograft model)

The following parameters were used to quantify the antitumor effects upon different treatments as previously described [33, 34]: (1) tumor volume measured during and at the end of experiments, (2) tumor weight measured at the end of experiment, (3) tumor progression (TP or doubling time) defined as an increase of greater than 100% of tumor volume with respect to baseline, and (4) time to progression (TTP) defined as the time for tumor progression.

### Orthotopic intra-brain model

Female CD1 nu/nu mice were inoculated intra-cerebrally as previously described [33, 34] with luciferase-transfected U251 and patient-derived GBM stem cell line (GSCs-5). Just before treatment initiation (5 days after injection), animals were randomized to treatment groups of 10 mice each. In vivo bioluminescence images were obtained using the UVITEC Cambridge Mini HD6 (UVItec Limited, Cambridge, UK). Animals were anesthetized, and luciferin (150 mg/kg) was injected intra-peritoneally (IP) 15 min prior to imaging. The mice were photographed while placed on their front, and the bioluminescence intensity (BLI) was measured in the region of interest. We deliberately inoculated a small number of cells (3 × 10^3^) to simulate a chemo-radio-therapeutic treatment made after surgery in which a low number of tumor cells, remaining the wound bed, and re-grow resulting in a recurrence. Treatments were started 5 days after cell injection when no luciferase activity was intracranially detectable. Mice were euthanized when they displayed neurological signs (e.g., altered gait, tremors/seizures, lethargy) or weight loss of 20% or greater of pre-surgical weight.

### Statistics

Continuous variables were summarized as mean and SD or as median with 95% CI. For continuous variables not normally distributed, statistical comparisons between control and treated groups were established by carrying out the Kruskal-Wallis tests. When Kruskal-Wallis tests revealed a statistical difference, pair-wise comparisons were made by Dwass-Steel-Chritchlow-Fligner method and the probability of each presumed “non-difference” was indicated. For continuous variables normally distributed, statistical comparisons between control and treated groups were established by carrying out the ANOVA test or by Student *t* test for unpaired data (for two comparisons). When the ANOVA revealed a statistical difference, pair-wise comparisons were made by Tukey’s HSD (honestly significant difference) test and the probability of each presumed “non-difference” was indicated. Dichotomous variables were summarized by absolute and/or relative frequencies. For dichotomous variables, statistical comparisons between control and treated groups were established by carrying out Fisher’s exact test. For multiple comparisons, the level of significance was corrected by multiplying the *P* value by the number of comparisons performed (*n*) according to Bonferroni correction. TTP was analyzed by Kaplan-Meier curves and Gehan’s generalized Wilcoxon test. When more than two survival curves were compared, the log-rank test for trend was used. This tests the probability that there is a trend in survival scores across the groups. All tests were two-sided and were determined by Monte Carlo significance. *P* values < 0.05 were considered statistically significant. SPSS® (statistical analysis software package) version 10.0 and StatDirect (version. 2.3.3., StatDirect Ltd) were used for statistical analysis and graphic presentation.

## Results

First, glioma cell models were grouped for MGMT expression levels. As previously described SF268, T98G, U138, U118, LN18, D54, and SW1783 show high levels of MGMT, whereas U251, U87, A172, U373, SNB19, and LN229 show low or absent levels due to complete or hemi-methylation of MGMT gene [36–39]. Seven GBM patient-derived stem cell lines were characterized as MGMT positive (BT12M, BT25M, BT50EF, and CSC-7) and negative (BT48EF BT53M and CSCs-5) [39].

### Antitumor effects of TINO in glioma cell models: comparison with BDM and SAHA alone or in combination

**Fig. 1:**
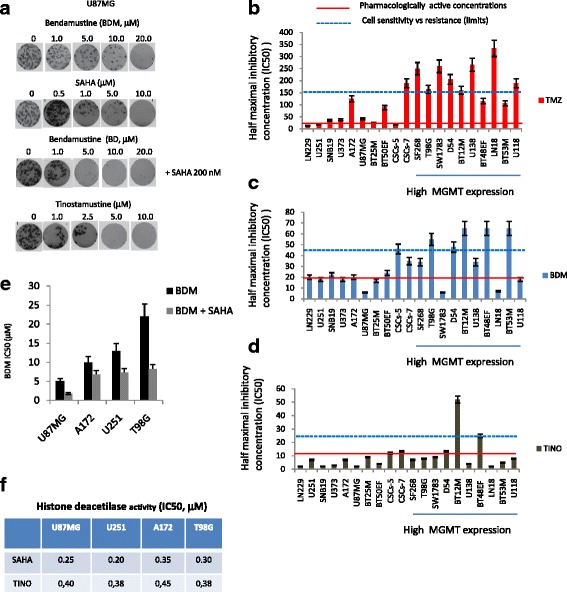
Antitumor effects of TINO in glioma cell models: comparison with BDM and SAHA alone or in combination. **a** Representative crystal violet stain assay performed in U87MG cells. **b** IC50 values for temozolomide (TMZ) in different glioma and stem-like cells. **c** IC50 values for Bendamustine (BDM) in different glioma and stem-like cells. **d** IC50 values for tinostamustine (TINO) in different glioma and stem-like cells. In **b**, **c**, and **d** were added two markers: a red line showing the pharmacologically active concentration measured at the blood peak in humans for temozolomide (35 μM) and bendamustine (20 μM) as well as in brains of mice for tinostamustine (11.2 μM in the CNS after single i.v. bolus injection of 40 mg/kg, Huan Tun and collaborators manuscript submitted) and a dashed blue line indicating the in vitro cell sensitivity/resistance to the drugs. **e** Comparison on BDM and combination treatments using a fixed nontoxic dose of SAHA (200 nM) and increasing concentrations of BDM in U87MG, U251, A172, and T98G cell lines. **f** Histone deacetylase activity: comparison of inhibitory effects of SAHA and TINO. Single results are representative of three different experiments performed in triplicate

Initially different concentrations of BDM and TMZ were tested for inhibition of cell proliferation in our cell cohort. In Fig. 1a, we show the representation of crystal violet stain assay performed in U87MG cells. MTT was used to calculate the inhibition concentration at 50% (IC50) values. This assay was also used to compare the effects of temozolomide (Fig. 1b), bendamustine (Fig. 1c), and tinostamustine (Fig. 1d) in the different cell lines. Bendamustine (BDM) showed IC50 values ranging between 5.5 and 65.3 μM. Conversely, the majority of the cytotoxic effects caused by TMZ occurred between 12 and 334 μM. Interestingly, BDM was found to retain its cytotoxic activity when tested both against MGMT negative and TMZ-resistant cell lines (22.6 ± 10.9 μM [mean ± SD] versus 36.4 ± 21.8 μM, respectively *P* = 0.0968 [NS]) In contrast, the effects of TMZ were strongly dependent on MGMT expression (73.4 ± 20.1 μM in MGMT negative cells versus 190.7 ± 29.4 μM, in MGMT positive cells *P* = 0.0187). The effects of TINO were tested in the same cell systems: Based on IC50 values, TINO was found to be amongst the most potent agents tested with a range of 1.7 μM and 52.0 μM (6.1 ± 1.3 μM in MGMT negative versus 13.3 ± 4.8 μM in MGMT positive, *P* = .1629 NS). All cell lines, including 5/7 GSC lines resulted in a moderately/highly sensitive IC50 ranged between 4.3 and 13.4 μM and 2/7 GSC showed IC50 > 25 μM. These data suggest a TINO IC50 of a 5 to 25 order of magnitude lower than for BDM. The effects of TINO were compared with a combination treatment using a fixed nontoxic dose of SAHA (200 nM) and increasing concentrations of BDM in U87MG, U251, A172, and T98G cell lines. The IC50 values decreased significantly in the four cell systems suggesting that SAHA was a chemo-sensitizing compound in glioblastoma cells (Fig. 1e). We verified by HDAC activity assay that TINO maintained the HDAC inhibition activities of SAHA in the acetylation status of histone H3 (Fig. 1f). Similar inhibition of H3 histone deacetylation by SAHA and TINO was shown in U87MG cells. Similarly, SAHA and TINO were able to modulate differentiation status of U87MG cells, including the expression of MGMT. GFAP, nestin, and β-catenin proteins were also similarly modulated by SAHA and TINO at IC50 values (data not shown) suggesting that TINO maintained the differentiation properties of SAHA.

### Tinostamustine increases the effects of radiotherapy in glioma models in vitro

**Fig. 2:**
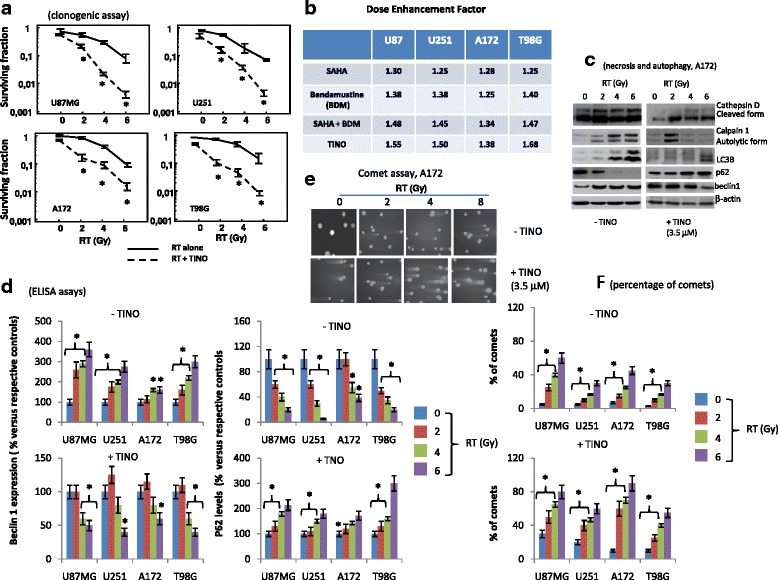
Tinostamustine increases the effects of radiotherapy in glioma models in vitro (part I). **a** Clonogenic assay performed on U87MG, U251, A172, and T98G cells showing radio-sensitizing effects of tinostamustine (TINO). **b** Comparison between dose enhancement factor (DRE) calculated in U87MG, U251, A172, and T98G in cultures treated with Rt combined to SAHA, BDM, SAHA + BDM, and TINO and showing higher DRE values in the combination of TINO and RT. **c** Western blots for autophagy and necrosis marker expression in A172 cells used as representative cell line for this analyses. **d** ELISA determination of beclin 1 and p62 proteins in U87MG, U251, A172, and T98G cells. ELISA determinations performed in quintuplicate. Mean values (mean values ± standard errors, SD) were transformed as % versus relative controls. **e** Representative comet assay obtained in A172 cells treated with different doses of RT with or without 3.5 μM TINO. **f** Quantification of comet assays as percentage of comets (with intermediated/high comet tails) in U87MG, U251, A172, and T98G; Single results are representative of three different experiments performed in triplicate. Statistical analyses: *p < 0.005

**Fig. 3:**
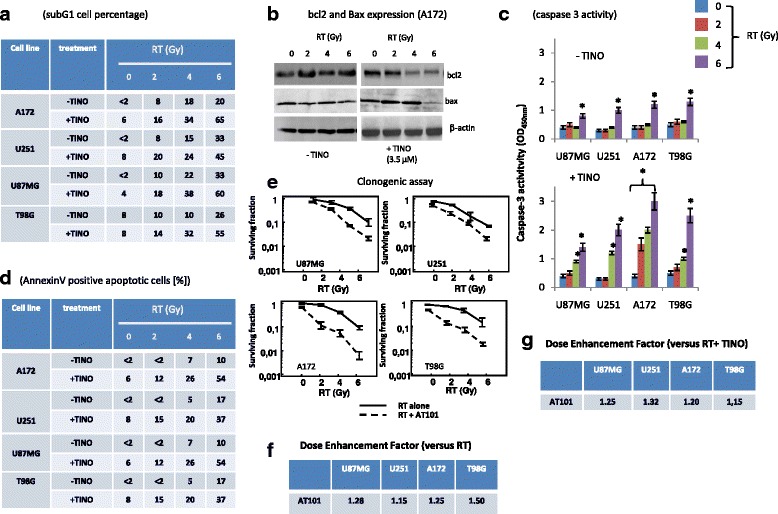
Tinostamustine increases the effects of radiotherapy in glioma models in vitro (part II). **a** Table summarizing subG1 cell percentage measured with propidium iodide (PI) by using Tali™ instrument (Thermo Fisher Scientifics, Carlsbad, CA, USA) in U87MG, U251, A172, and T98G treated with RT with or without TINO. **b** Western blot performed on Bcl2 and Bax expression performed in A172 cells treated with RT (0–6 Gy) with or without TINO 3.5 μM. **c** Caspase 3 activity measured in U87MG, U251, A172, and T98G treated with RT (0–6 Gy) with or without TINO. **d** Annexin V/PI positive apoptotic cells performed by using Tali™ Apoptosis Kit - Annexin V Alexa Fluor™ 488 & Propidium Iodide. **e** Clonogenic assay performed with AT101 (Gossypol, 10.0 μM), Bcl2 inhibitor in U87MG, U251, A172, and T98G. **f** Table summarizing DRE values calculated in U87MG, U251, A172, and T98G treated with RT (0–6 Gy) with or without 10.0 μM AT101. **g** Table summarizing DRE values calculated in U87MG, U251, A172, and T98G treated with RT (0–6 Gy) with or without 10.0 μM AT101 and TINO 3.5 μM (triple combination). Single results are representative of three different experiments performed in triplicate

To determine the effects of TINO on the radio-sensitivity of GBM cells, a clonogenic assay was performed. As drug exposure times are longer during clonogenic survival studies with radiation, 1 μM TINO was considered. This value was lower than the IC20 value for the majority of GBM cell lines. The in vitro analyses were performed on 4/12 high-grade glioma cell lines (U87MG, U251, A172, and T98G) chosen as models for molecular and functional studies. The choice on these models resulted from the necessity to have sensitivity scale versus RT and TINO administration. Treatment of glioma cells with 1 μM TINO alone yielded a surviving fraction of 0.90 ± 0.064 in U251, 0.77 ± 0.18 in U87, 0.94 ± 0.036 in T98G, and 0.85 ± 0.12 in A172. Tinostamustine induced a strong radio-sensitizing effect in our cell models (Fig. 2a). In the combination protocol 24–48 h after drug administration, cells were irradiated at 2, 4, and 6 Gy and colony forming efficiency was determined 30 days later. Clonogenic assay (Fig. 2b) demonstrates that this combination treatment resulted in a dose enhancement factor (DRE) of 1.50 in U251, 1.55 in U87MG, 1.38 in A172, and 1.68 in T98G cells. Next, we evaluated the changes in apoptotic, autophagic, and necrotic proteins in our four cell models. In agreement with the previous data demonstrating that in glioma cells, RT-associated cell death was mainly a mixture of necrosis and autophagy [40], we show a dose-dependent increase in cathepsin D cleavage and calpain 1 autolysis (necrosis). Similarly, a dose-dependent increment of levels of LC3B, for cleavage of LC3 protein, and beclin 1 associated with a decrement or loss of p62 protein were observed (autophagy). In addition, we demonstrated that the administration of TINO, administered at IC50 values reduced LC3B and Beclin 1 levels as well as slowed/impeded the down-modulation of p62. In Fig. 2c, we show western bots obtained in representative A172 cell extracts. Similar results were obtained in U87MG, U251, and T98G cells. Next, p62 and beclin1 levels were quantified by ELISA determinations performed in quintuplicate. Mean values (mean values ± standard errors, SD) were transformed as % versus relative controls. These results are shown in Fig. 2d. Comet assay were performed on culture treated with different doses of RT with or without 3.5 μM TINO. In Fig. 2e, we show representative data obtained on A172 cells. We demonstrated that the co-administration of TINO amplified the DNA damage (comets) induced by RT. Similar results were obtained in U87MG, U251, and T98G. In Fig. 2f, we quantified the comets as percentage of cells with comets analyzed in our four cell models of glioma. All together, these results were associated with a strong increase of sub-G1 cell percentage obtained by FACS (Fig. 3a). In Fig. 3b, we show that Bcl2 and Bax levels were weakly upmodulated after RT treatment. TINO was able to reduce Bcl2 and maintain high Bax expression when administered in combination with RT doses suggesting a possible replacement of autophagy in this combinatorial therapy. In agreement with these data, we found that caspase 3 activity was significantly increased after TINO administration (Fig. 3c). A further demonstration of Bcl2 in increased apoptosis was evident by the comparison of the percentage of annexin V/PI-positive cells (apoptosis, Fig. 3d) versus the total subG1 cell population (mixed cell death) in RT and RT + TINO treatments. Next, we analyzed the role of Bcl2 in this phenomenon by using the bcl2 inhibitor AT101 at dose of 5.0 μM [41]. We observed that also AT101 showed in glioma cell models strong radio-sensitizing effects in agreement with our previous experiments [42]. In Fig. 3e, we show the clonogenic curves for our four cell models. The analyses of dose enhancement factor values (Fig. 3f) showed, however, lower radio-sensitizing effects to those observed for TINO. When AT101 was co-administered with the combination TINO + RT, the clonogenic curves showed a further dose enhancement factor as indicated in Fig. 3g. This increase was, however, significantly low due of high AT101 dose used for this triple pharmacological combination.

**Fig. 4:**
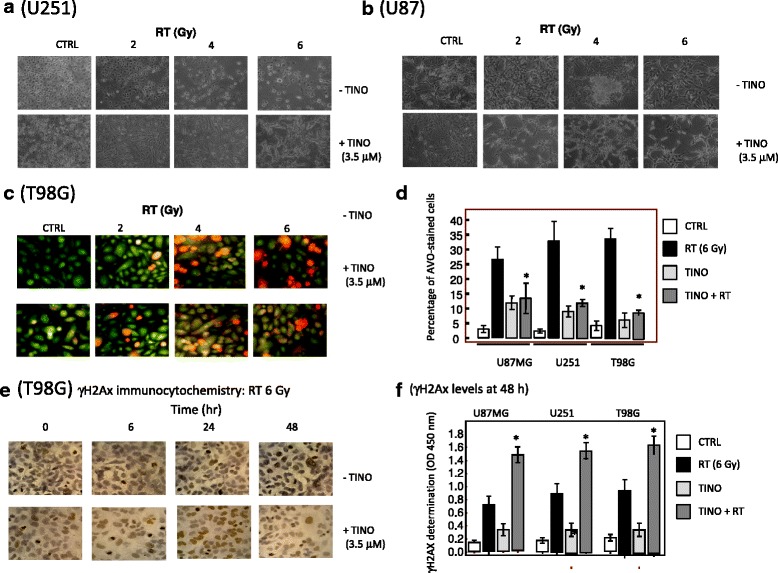
Morphological and functional effects of TINO when combined with RT in glioma cell lines. **a** Morphological changes observed in U251 cells after treatments with 2, 4, and 6 Gy (RT) alone or in association with TINO 3.5 μM. **b** Morphological changes observed in U87MG cells after treatments with 2, 4, and 6 Gy (RT) alone or in association with TINO 3.5 μM. **c** Representative expression of acidic vacuolar organelle (AVO) accumulation in T98G cells cultured with 2, 4, and 6 Gy (RT) alone or in association with TINO 3.5 μM. **d** Percentage of AVO-stained cells in U87MG, U251, and T98G cells cultured with 2, 4, and 6 Gy (RT) alone or in association with TINO 3.5 μM. Statistical analysis: *p < 0.005 in the comparison between combined TINO + RT treatment and RT alone. Single results are representative of three different experiments performed in triplicate. **e** Immunocytochemical evaluation of γH2Ax stain in T98G cells cultured with 4, 6, and 8 Gy (RT) alone or in association with TINO 3.5 μM. **f** Quantization of γH2AX expression in U87MG, U251, and T98G cells treated with RT with or without TINO. Statistical analyses: *p < 0.005 in the comparison between RT + TINO and RT or TINO treatments alone. Single results are representative of three different experiments performed in triplicate

When combined with RT, TINO induced deep morphological changes that were evident in GBM cell cultures (Fig. 4a) (U251) and 4B (U87MG). In our experiments with TINO and or RT, we find that the morphology of U87MG passed from one messy appearance of cell colony to an intermediated epithelial like/fibroblastoid and bipolar nature morphology. In the old version of figure, I had chosen a more dense control for TINO administration to show the morphology change at sub-confluence. A further demonstration of reduced necrosis after administration of TINO to RT-treated cell cultures was evident for T98G cells after staining with acridine orange and propidium iodide (Fig. 4c). The presence of orange/red stained cells due to the accumulation of acidic vacuolar organelles (AVO) was elevated with RT in a dose-dependent manner. The addition of TINO reduced the presence of AVO in treated cells. The percentage of AVO-stained cells on five different 10× stained microscopic field in U87MG, U251, and T98G cells was associated with increased γH2Ax staining (Fig. 4d). In Fig. 4e, T98G cultures stained for γH2Ax at 24 h after RT administration alone or combined with TINO. In Fig. 4f, U87MG, U251, and T98G cell cultures were treated with RT alone or in combination with TINO and levels of γH2Ax were quantified by immuno-enzymatic colorimetric assay. The levels of γH2Ax were elevated in combined treatments after 24 h from start of experiment. In agreement with literature data, RT administration caused H2Ax phosphorylation, and γH2AX levels were maximal within 4–6 h, reaching baseline values within 12 h. Tinostamustine-induced maximal γH2AX levels were observed within 2–4 h with a slower decrease in the time and an achievement of baseline values within 24–48 h. When TINO was combined with RT, the levels of γH2AX were higher within 2–4 h and the time necessary to reach baseline values of γH2AX was slightly increased (data not shown) suggesting that DNA repair was impaired (reduced or retarded).

**Fig. 5:**
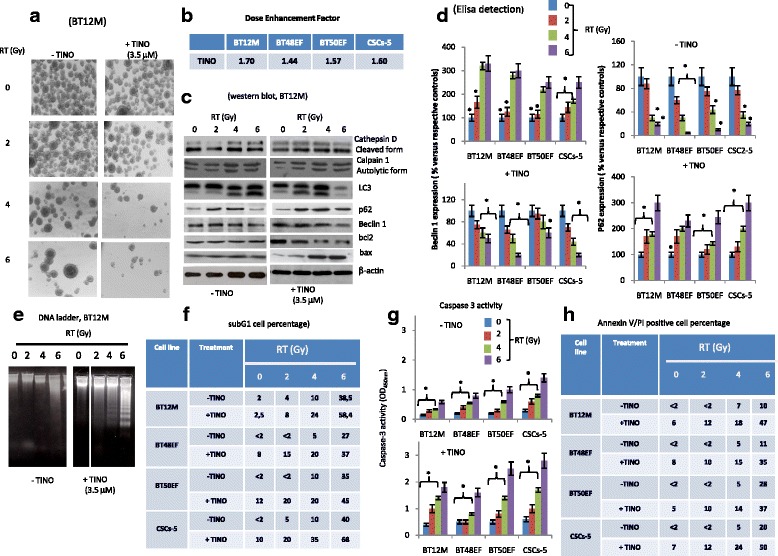
Effects of TINO with or without RT in patient derived glioma stem-like cells. **a** Effects of RT (0, 2, 4, and 6 Gy) with or without TINO in BT12M cell model. **b** DRE values calculated for BT12M, BT48EF, BT50EF, and CSCs-5 patient-derived glioma stem-like cells. **c**, **d** Western blot and ELISA determinations indicating the shift from authophagy to apoptosis. **e** DNA ladder in representative BT12M cells. **f** Table summarizing subG1 cell percentage measured with propidium iodide (PI) by using Tali™ instrument (Thermo Fisher Scientifics, Carlsbad, CA, USA) in BT12M, BT48EF, BT50EF, and CSCs-5 patient-derived glioma stem-like cells. **g** Caspase 3 activity performed in BT12M, BT48EF, BT50EF, and CSCs-5 patient-derived glioma stem-like cells. **h** Annexin V/PI-positive apoptotic cells performed by using Tali™ Apoptosis Kit - Annexin V Alexa Fluor™ 488 & Propidium Iodide. Single results are representative of three different experiments performed in triplicate

Similar experiments were repeated by using the patient-derived glioma stem cell cultures. Evaluation of RT in combination with IC20 TINO (8 μM) in 4/7 glioma stem-like (BT12M, BT48EF, BT53EF, and CSCs-5) cells demonstrated a reduction in the number of colonies compared with RT alone (Fig. 5a, BT12M cell model). Clonogenic curves were generated considering the number of colonies made up at 21 days of cultures and DRE calculated for our cell models and data shown in Fig. 5b. DRE values were significantly higher compared to those observed for glioma cell lines (see Fig. 2b) and ranged between 1.44 (BT50EF) and 1.70 (BT12M). This was associated with increased apoptosis and a lower contribution of necrosis as suggested by (i) western blot and ELISA determinations (Fig. 5c, 5d) showing a reduction of RT-mediated expression of LC3B and Beclin 1 and induction of p62), (ii) DNA ladder (Fig. 5e, representative BT12M cells), (iii) BCl2 down-modulation after tinostamustine administration (Fig. 5c), and (c) increased caspase 3 activity (Fig. 5g for all four cell models). Low contribution of autophagy in cell death was also suggested analyzing the differences of percentage of sub-G1 cell distribution (Fig. 5f) and annexin V/PI positive (Fig. 5h) cells.

### In vivo efficacy of TINO alone or in combination with RT (subcutaneous xenograft model)

**Fig. 6:**
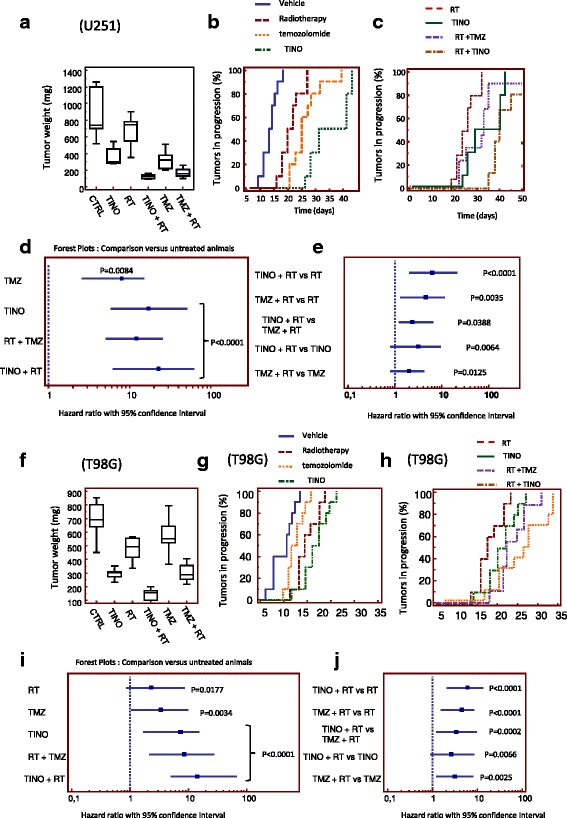
Radiosensitizing effects of TINO on tumor weight and time to progression in U251 and T98G xenograft models. **a** Analysis of tumor weights harvested at the end of experiment (50th day from randomization) in U251MG xenografts. **b** Kaplan-Meier curves generated for time to progression in U251: comparisons amongst RT, TMZ, and TINO single therapies. **c** Kaplan-Meier curves generated for time to progression: analysis of radio-sensitizing effects of TINO for U251G xenografts in comparison with TMZ and RT + TMZ. **d** Forest plots (U251) for the comparison of TTP distribution in different treatments with untreated animals (vehicle). **e** Forest plots (U251) for comparison of TTP in the different combination regimens. **f** Analysis of tumor weights harvested at the end of experiment (35th day from randomization) in T98G xenografts. **g** Kaplan-Meier curves generated for time to progression in T98G: comparisons amongst RT, TMZ, and TINO single therapies. **h** Kaplan-Meier curves generated for time to progression: analysis of radio-sensitizing effects of TINO for T98G xenografts in comparison with TMZ and RT + TMZ. **i** Forest plots for the comparison of TTP distribution in different treatments with untreated animals (vehicle). **j** Forest plots for comparison of TTP in the different combination regimens

To assess the effect on tumors in an in vivo model, 1 × 10^6^ cells of U251, U87MG, and T98G GBM cells were subcutaneously injected in female cd1 nu/nu mice. The changes in tumor volumes were measured in order to calculate the TTP. At the end of the therapy cycle (35–50 days), animals were sacrificed and the tumor harvested, weighed, and processed for molecular and histochemical analyses. Tinostamustine monotherapy demonstrated an anti-tumor effect reducing tumor weight by 40, 56, and 60% in U251 (Fig. 6a), U87MG (Additional file 1: Figure S1A), and T98G (Fig. 6f) xenografts, respectively. TINO also increased the antitumor effects of RT reducing tumor weight by 82, 80, and 76% (compared with vehicle) in U87MG, U251, and T98G xenografts, respectively. Since RT alone reduced tumor weight by 20, 22, and 33% in the same cell models, a resultant combination index [CI] can be calculated for each xenograft: 0.73 in U87MG, 0.97 in U251, and 0.87 in T98G. These CI values suggest additive/synergy between TINO and RT. Temozolomide showed efficacy similar to TINO monotherapy in U87MG and U251 cells, with final tumor weight reductions of 53 and 54% compared with controls, whereas a very low effect (12% reduction versus control) was observed in T98G cells. Temozolomide used at 16 mg/kg (equivalent human dose for mice [43]) also increased RT sensitivity, with tumor weight reductions (compared with vehicle control) of 83% (CI = 0.85), 71% (CI = 0.74), and 57% (CI = 0.89), in U87MG, U251, and T98G, respectively. CI values for TMZ were also in the range of synergism. The TTP was plotted versus time to generate Kaplan-Meier curves showed a significant antitumor effect with TINO monotherapy (Fig. 6b, 6f and Additional file 1: Figure S1B for U251, T98G, and U87 cell xenografts, respectively) was superior to those observed for RT and TMZ mono-therapies.

The combination with RT and TMZ or TINO showed higher radio-sensitizing effects of TINO than TMZ (Fig. 6c, g and Additional file 1: Figure S1C for U251, T98G, and U87 cell xenografts, respectively). Tinostamustine showed similar effects to the gold standard in all cell models. However, a forest plot graphical representation of this meta-analysis demonstrates greater effects (higher hazard ratios (HR)) with TINO than RT and TMZ as single treatments. Similarly, TINO showed greater RT sensitizing effects with higher HRs when combined with RT compared with the RT plus TMZ combination (Fig. 6c, 6d; Fig. 6h–6l; Additional file 1: Figure S1C, D and E, in U251, T98G, and U87MG cell xenografts, respectively). The combination TINO plus RT was more effective than the gold standard (RT plus TMZ) in all cell xenografts with HR = 0.94, 2.35, and 3.39 in U251, U87MG, and T98G xenografts, respectively, suggesting a higher effects in MGMT+/TMZ resistant T98G cells. Additional file 2: Table S1 summarized the statistical analysis for the Kaplan-Meier curves showed in Additional file 1: Figure S1 and Fig. 6.

### Histopathological and immunohistochemical effects of treatment with RT and/or tinostamustine

**Fig. 7:**
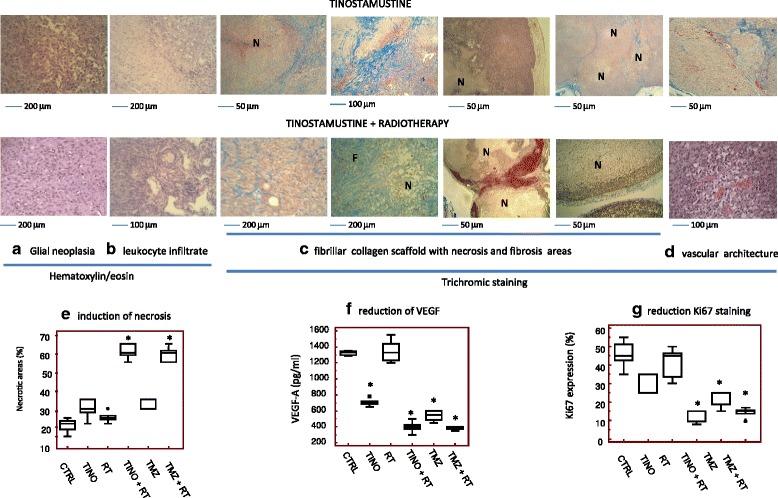
Representative histopathological and immunohistochemical images in T98G tumors treated with TINO and TINO + RT. **a**, **b** Hematoxylin/eosin staining for glial appearance of T98G tumors and leukocyte infiltrate. **c** Different pattern of fibrillar collagen scaffold with necrosis and fibrosis areas evaluated by trichromic stain. **d** Modification of vascular architecture in TINO and RT + TINO treatments. **e** Determination of necrotic areas in T98G tumors and comparison amongst experimental groups. **f** VEGF-A determination on plasma collected from mice bearing T98G tumors and comparison amongst experimental group. **g** Ki67 staining (proliferating index) in T98G tumors harvested from different experimental group

Histologically, U87MG, U251, and T98G tumors show glial morphology associated to high tumor cell rate and elevated cell pleomorphism. In Fig. 7a, we show this appearance for T98G. A narrow band of leukocyte infiltrate, consisting of granulocytes, B lymphocytes, and monocyte/macrophages may surround the tumors and constitute a specialized neoplastic microenvironment (T98G, Fig. 7b). Tumor cells are dispersed a fibrillar collagen background that may envelop abundant vasculature (Fig. 7c, 7d). A pseudopalisading necrosis was also evident in addition to thrombotic vessels and hemorrhage. Necrosis and fibrosis are increased in the combination treatment with TINO and RT when compared to tinostamustine administration or RT treatment alone. In Fig. 7e, we show the analysis on necrosis (percentage or necrotic areas in 10 separate histological fields in T98G tumors comparing our treatments with the standard therapies (temozolomide and temozolomide associated with RT). The percentage of necrotic areas was significantly higher in TINO + RT treatment compared to TINO and RT alone and similar to those observed for the treatment with temozolomide and RT. This was associated with reduced areas with high tumor proliferation rate increasing the percentage of areas with stroma (Fig. 7c, 7d). Elevated percentage of necrotic regions could be due to an inadequate blood supply as a response to treatment. So, the expression of VEGF was evaluated in the blood of mice bearing T98G tumors (Fig. 7d). Increased fibrosis and reduced angiogenesis are related to reduced Ki67 as shown for U87MG xenograft in Fig. 7g.

### Orthotopic models: luciferase-transfected GBM cells

**Fig. 8:**
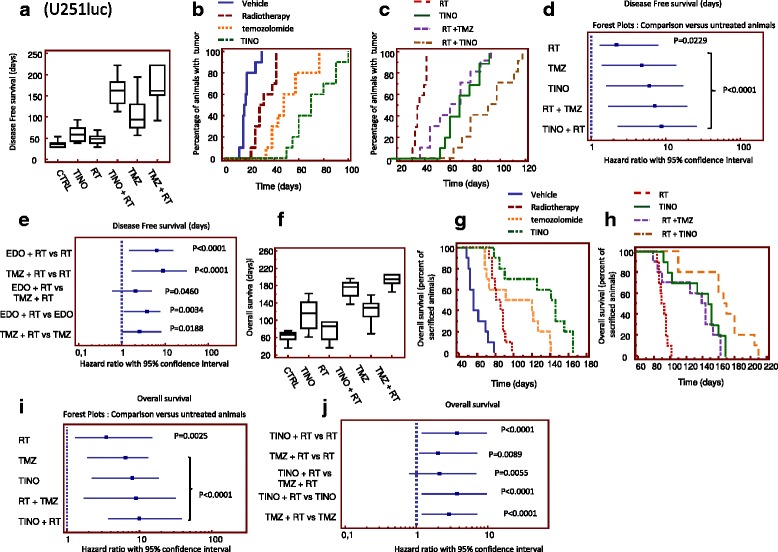
Orthotopic intra-brain injection of differentiated U251 cells. **a** Graphical analyses for the time of time to detection of bioluminescence which is related to Disease Free survival (DFS) parameter: Median ± CI 95% DFS data (days) for each treatment. **b** Kaplan Meyer curves generated for tumor detection in animal of control or treated with single treatments (RT, TINO and TMZ). **c** Kaplan Meyer curves generated for DFS data in animal treated with RT, TINO and their combination. Comparison with standard chemo-radiotherapy consisting in the combination of RT and TMZ. **d** Forest plots for the comparison of DFS with untreated animals (vehicle treated). **e** Forest plots for comparison of DFS in combination regimens. **f** Graphical analyses for the time of overall survival (OS): median ± CI 95% OS data (days) for each treatment. **g** Kaplan-Meier curves generated for OS in animal of control (Vehicle), or treated with RT, TINO and TMZ for the comparison of single treatments. **h** Kaplan-Meier curves generated for OS in animal treated with RT, TINO, and their combination. Comparison with standard chemo-radiotherapy consisting in the combination of RT and TMZ. **i** Forest plots for the comparison of OS with untreated animals (vehicle treated). **j** Forest plots for comparison of OS in combination regimens

The efficacy of TINO was investigated in an orthotopic mouse model using differentiated luciferase-transfected GBM cells (U251). A small number of cells (3 × 10^3^) were inoculated deliberately to simulate treatment post-surgery. Treatments were started 5 days after cell injection when no luciferase activity was detectable intracranially; the animals were treated for 35 days and followed for a maximum of 220 days. Disease-free survival (DFS, Fig. 7a) was the time without brain bioluminescence, and overall survival (OS, Fig. 7f) as the time necessary to observe clinical signs of distress ethically considered for the sacrifice of animals. As first analyses, we evaluated the recurrence time defined as the time in which a luciferase activity was intracranially detectable. Untreated animals showed bioluminescence signals after about 30 days (35.0 ± 2.7; mean ± SEM). RT increased DFS, slowing mean recurrence to 40.0 ± 3.8 days (Fig. 7a). Mean recurrence was also significantly slowed with TINO (55.0 ± 6.3) and TMZ (115 ± 16.4) treatments. Combination with RT further slowed time of recurrence by 120 ± 13.7 days for TINO and 105 ± 14.2 for TMZ. The comparison between TINO + RT and TMZ + RT combinations plus RT was not significant. Kaplan-Meier curves demonstrated that TINO monotherapy reduced tumor progression compared with untreated animals (Figs. 7b and 8d) with results superior to RT and TMZ alone though not significantly greater than RT + TMZ. Statistically significant differences in the radio-sensitizing effects of TINO versus TMX were observed in this radio- and chemo-sensitive model (Fig. 7c, 7e). In U251 orthotopic intra-brain xenograft, untreated animals showed distress signs and were sacrificed after about 50 days (55.0 ± 3.6). RT increased OS, slowing mean recurrence to 70.0 ± 7.3 days. Mean recurrence was also significantly slowed with TINO (120.0 ± 11.6) and TMZ (120 ± 8.7) monotherapies. Combination with RT further slowed time of recurrence to 180 ± 7.1 days for TINO plus RT which was significantly greater than for TMZ plus RT: 160 ± 4.8 for. Kaplan-Meier curves showed that TINO monotherapy increases OS with a HR of 6.6 compared with untreated animals (vehicle) (Fig. 7g, 7i). TINO was superior to RT and TMZ as single therapies and was significantly more effective in comparison with RT plus TMZ (Fig. 7b, 7e). Statistically significant differences in the radio sensitizing effects of TINO versus TMZ were observed in this radio- and chemo-sensitive model (Fig. 7c, 7e). The same treatments were applied to orthotopic intra-brain xenografts originated from patient-derived GBM stem-like cells, CSCs-5 previously transfected by luciferase. In this cell system, untreated animals showed a bioluminescent positivity after about 10-20 days (16.5 ± 1.7; mean ± SEM). RT increased DFS up to 29.5 ± 2.3 days (Fig. 8a). Mean DFS was also significantly increased with TINO (81.0 ± 7.4) and TMZ (62.0 ± 8.1) treatments. TMZ plus RT showed a DFS of 82.0 ± 15.7 days whereas the TINO plus RT combination was significantly greater at 144.0 ± 15.9 days (*p* < 0.0005). TINO monotherapy reduced tumor progression on Kaplan-Meier analysis compared with untreated animals (Fig. 8b, 8d). TINO was superior to RT and temozolomide as single treatments. TINO in combination with RT further reduced tumor progression compared with vehicle (#Fig. 8b, 8d) and either RT or TINO (Fig. 8c, 8e). Statistically significant differences in the radio-sensitizing effects of TINO versus TMZ in this radio- and chemo-sensitive model were observed (Fig. 8e) suggesting that TINO shows higher radio-sensitizing effects than TMZ. Untreated animals showing distress signs and were sacrificed after about 50 days (48.0 ± 3.6). RT increased OS, slowing mean recurrence to 80.0 ± 4.8 days (Fig. 8f). Mean DFS was also significantly increased with TINO (140.0 ± 23.5) and TMZ (105.5 ± 16.5) treatments. Combination with TMZ and RT showed a DFS of 145.0 ± 22.5 days whereas the combination RT with TINO reached 210.0 ± 35.0 days.

**Fig. 9:**
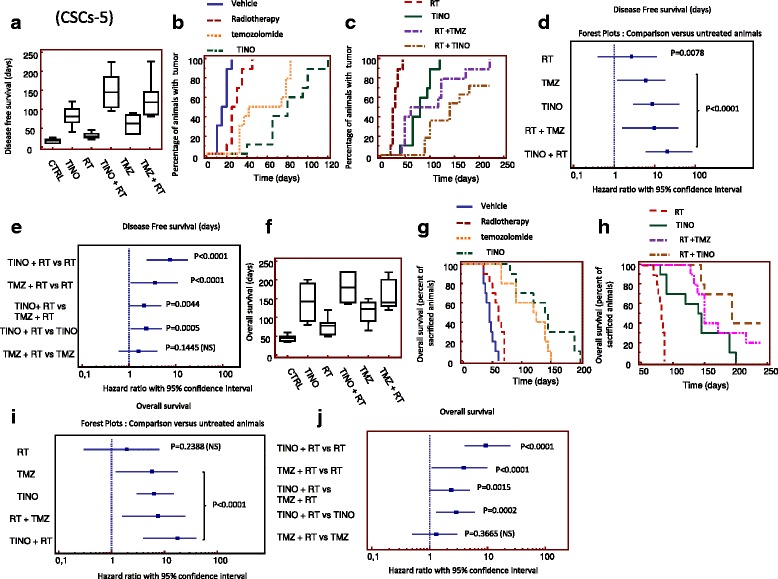
Orthotopic intra-brain injection of patient-derived GBM stem-like cells (CSCs-5) and comparison with different treatment groups. **a** Graphical analyses for the time of Time to detection of bioluminescence or DFS: Median ± CI 95% DFS data (days) for each treatment. **b** Kaplan-Meier curves generated for tumor detection in animal of control or treated with single treatments (RT, TINO and TMZ). **c** Kaplan Meyer curves generated for DFS data in animal treated with RT, TINO and their combination. Comparison with standard chemo-radiotherapy consisting in the combination of RT and TMZ. **d** Forest plots for the comparison of DFS with untreated animals (vehicle treated). **e** Forest plots for comparison of DFS in combination regimens. **f** Graphical analyses for the time of overall survival (OS): Median ± CI 95% OS data (days) for each treatment. **g** Kaplan-Meier curves generated for OS in animal of control (Vehicle), or treated with RT, TINO, and TMZ for the comparison of single treatments. **h** Kaplan Meyer curves generated for OS in animal treated with RT, TINO, and their combination. Comparison with standard chemo-radiotherapy consisting in the combination of RT and TMZ. **i** Forest plots for the comparison of OS with untreated animals (vehicle treated). **j** Forest plots for comparison of OS in combination regimens

Analyzing the Kaplan-Meier curves obtained plotting the percentage of sacrificed animals in the time; we observe that TINO monotherapy increases OS with a HR of 5.8 with respect to vehicle, compared with untreated animals (Fig. 8g, 8i). TINO was superior to RT and TMZ as single therapies. TINO in combination with RT further increases OS compared with vehicle (Fig. 8g, 8i), RT (Fig. 8h, 8j), or TINO (Fig. 8h, 8j). Statistically significant differences in the radio-sensitizing effects of TINO versus TMZ were also observed in this radio- and chemo-sensitive model suggesting that TINO show higher radio-sensitizing effects than TMZ. Additional file 2: Tables S2 and S3 summarizes statistical analyses of Figs. 8 and 9.

## Discussion

High-grade gliomas represent the most aggressive primary brain tumors showing high resistance to classic cytotoxic therapies. DNA damage triggers a series of signaling cascades promoting cell death as well as cellular survival, including DNA repair, cell cycle arrest, and autophagy. The elevated basal and/or stressful levels of both DNA repair and autophagy observed in tumor cells, in contrast to normal cells, have been identified as the most important drug-responsive programs that impact the outcome of anticancer therapy. For over a decade, RT and TMZ combination therapy has been the gold standard with clinical trial of alternatives being unsuccessful [1, 3]. However, the prognosis remains poor and some subgroups of patients, like those with MGMT unmethylated tumors do not benefit from this regimen [1, 8, 9] and therapeutic resistance in MGMT-unmethylated tumors has emerged as an important clinical issue. It is necessary to consider that 35 μM is the blood peak of concentration of TMZ when this compound was administered to humans [44, 45] and this concentration was active in 5 out 12 GBM cells lines and 2 out 7 patient-derived GBM proliferating/stem cell lines. BDM is distributed freely in blood, with a concentration at the peak ranging between 10 and 100 μg/ml (corresponding to 28–280 μM) [46, 47] and that plasma concentrations were approximately 100-fold higher than in mouse brain [48]. So in vitro concentrations of BDM are higher when compared with those observed in vivo. In addition, T98G and SW1783 cell lines, which are characterized by high levels of MGMT, showed a pronounced resistance to TMZ (TMZ IC50 of 147.2 ± 2.1 μM and 234.6 ± 2.3 μM, respectively), whereas A172 and LN229 cell lines, which exhibit no or low MGMT expression, had low TMZ IC50 values (14.1 ± 1.1 μM and 14.5 ± 1.1 μM, respectively). MGMT expression status had little effect on the activity of BDM, which demonstrated an antitumor effect in TMZ-resistant GBM cells (Fig. 1). The potential of SAHA [15, 49] and BDM [10, 11] as a target for radio-sensitization has been previously suggested in studies with other agents in experimental tumor models. In our report we test a new therapeutic strategy to improve the therapeutic outcome of combined radiotherapy with alkylating agents such as BDM by using tinostamustine (EDO-S101), a first-in-class alkylating deacetylase inhibitor (AK-DACi) molecule. This molecule fuses the DNA damaging effect of BDM with the fully functional pan-histone deacetylase (HDAC) inhibitor, vorinostat in a completely new chemical entity [25–27, 49]. We hypothesized that tinostamustine may induce radio-sensitization through different mechanisms associated with enhanced apoptosis despite a RT-induced autophagy. Target cells for these effects are both glioma cells and the glioma stem like cells (GSCs). One of the factors underlying tumor recurrence and poor long-term survival is, indeed, the presence of a cancer stem-like cell population GSCs which contribute to cancer invasion, angiogenesis, immune evasion, therapeutic resistance, and may drive recurrence [50–52]. The HDAC inhibitor vorinostat (SAHA) has received significant attention in recent years as an “epigenetic” drug used to treat solid tumors [53] which down-modulate cancer stem markers inducing differentiation of GSCs. The data presented here indicate that TINO increases DNA damage induced after RT, based on comet assay analyses and γH2Ax detection. Tinostamustine was able to reduce the expression of autophagy marker (LC3B and beclin1) increasing those of p62 suggesting a shift of cell death from autophagy to apoptosis. A further prove of this event is the reduction of levels of Bcl2 by TINO as well as the pharmacological inhibition of Bcl2 by AT101 (Gossypol). Reduced Bcl2 levels after co-administration of TINO to RT were translated both into an increased activity of caspase 3 (and an increased percentage of annexin V positive apoptotic cells. To further elucidate the role of Bcl2 in increased radio-sensitivity we performed clonogenic experiments on glioma cell cultures by administration of fixed 5 μM AT101. These experiments indicated a strong radio-sensitization of Bcl2 activity inhibition. The analyses of dose enhancement factors, calculated for the administration of AT101 with RT indicated the stronger effect in T98G (DRE = 1.50) and the lower effect in U251 (DRE = 1.15). A172 showed a DRE = 1.25 and U87MG a DRE = 1.28. This DRE values were compared with those observed for Tinostamustine. We found that the administration of AT101 determined similar DRE of Tinostamustine in U87MG and A172 with an increment of 2.4 and 1.6% in Tinostamustine treated cells, respectively. Tinostamustine was more active compared to AT101 in U251 (+ 13.6%) whereas AT101 was more active compared to tinostamustine in T98G (+ 20%) cells. This could be due to differences in basal Bcl2 expression levels observed in the different cell lines. When AT101 was administered with dual tinostamustine + RT (triple co-administration experiments) we noted an additive effect with increments in DRE values of 4% (U87MG), 5.6% (U251), 6.7% (A172) and 30% (T98G). This suggest that the efficacy of tinostamustine may be influenced by Bcl2 levels also if further studies should be necessary including Bcl2 transfection in low Bcl2 expressing glioma cell lines. Preliminary our data suggest that the single administration of Tinostamustine is more active in glioma cells with lower basal levels of Bcl2 (manuscript in preparation). Although the dose of 5.0 μM AT101, used for this analysis, was too high to evaluate a possible therapeutic approach, this was not a topic of the present report also if should be considered for further studies. Increasing evidence suggests that an inflammatory microenvironment may promote invasion by GBM cells [54, 55] through the activation of pathways that recruit myeloid precursors. One interesting possible mechanism involved in the sensitivity of GBM tumors to TINO and TINO plus RT is the recruitment of monocytes/macrophages. The possible involvement of proliferating monocytes is also invoked due to the presence of close “germinal cores/cluster” dispersed in the necrotic tumor masses. This event could have dual effects: to participate in the elimination of necrotic cells (resolution) or to mediate the awakening of quiescent stem cells (leading to recurrence). The latter effect was not supported by results in the orthotopic models, which demonstrated no increase in recurrence with TINO and the TINO plus RT combination after only 35 days of treatment (one treatment cycle). The rate of recurrence and the survival percentage in combination treatment were significantly better than those observed for the standard treatment, TMZ plus RT, providing evidence against recurrence due to stimulation of cancer stem cells.

## Conclusions

These data demonstrate that TINO is a broadly active antitumor agent in vitro and in vivo, with potent radio-sensitizing activity in aggressive and TMZ-resistant glioblastoma tumor models, supporting ongoing clinical evaluation of this compound in combination with RT for the treatment of post-surgery glioblastoma patients.

## Additional files

Additional file 1: **Figure S1.** Radiosensitizing effects of TINO on tumor weight and time to progression in U87MG xenograft model. (A) Analysis of tumor weights harvested at the end of experiment (50th day from randomization) in U251MG xenografts; (B) Kaplan-Meier curves generated for time to progression in U251: comparisons amongst RT, TMZ and TINO single therapies; (C) Kaplan-Meier curves generated for time to progression: analysis of radio-sensitizing effects of TINO for U251G xenografts in comparison with TMZ and RT + TMZ; (D) Forest plots (U87MG) for the comparison of TTP distribution in different treatments with untreated animals (vehicle); (E) Forest plots (U87MG) for comparison of TTP in the different combination regimens. (PDF 126 kb)

Additional file 2: **Table S1.** Additional hazard ratio analyses in xenograt models. Table S2 Additional hazard ratio analyses in orthotopic U251models. Table S3 Additional hazard ratio analyses in orthotopic CSCs-5model. (DOCX 23 kb)
