# Correction to: The first-in-class alkylating deacetylase inhibitor molecule tinostamustine shows antitumor effects and is synergistic with radiotherapy in preclinical models of glioblastoma

**DOI:** 10.1186/s13045-018-0587-3

**Published:** 2018-03-14

**Authors:** C. Festuccia, A. Mancini, A. Colapietro, G. L. Gravina, F. Vitale, F. Marampon, S. Delle Monache, S. Pompili, L. Cristiano, A. Vetuschi, V. Tombolini, Y. Chen, T. Mehrling

**Affiliations:** 1 Laboratory of Radiobiology, Department of Applied Clinical Sciences and Biotechnologies, University of L’Aquila, L’Aquila, Italy; 2 Laboratory of Radiobiology, Department of Applied Clinical Sciences and Biotechnologies, University of L’Aquila, L’Aquila, Italy; 3 Division of Radiotherapy, Department of Applied Clinical Sciences and Biotechnologies, University of L’Aquila, L’Aquila, Italy; 4 Division of Neurosciences, Department of Applied Clinical Sciences and Biotechnologies, University of L’Aquila, L’Aquila, Italy; 5 Division of Applied Biology, Department of Applied Clinical Sciences and Biotechnologies, University of L’Aquila, L’Aquila, Italy; 6 Division of Human Anatomy, Department of Applied Clinical Sciences and Biotechnologies, University of L’Aquila, L’Aquila, Italy; 7 Laboratory of Applied Biology, Department of Life, Health and Environmental Sciences, University of L’Aquila, L’Aquila, Italy; 8 Division of Radiotherapy, Department of Experimental Medicine, University of Rome “La Sapienza”, Rome, Italy; 9 Northlake International LLC, Pleasanton, CA USA; 10 Mundipharma-EDO GmbH, Basel, Switzerland

## Erratum

The original article [1] contained an error whereby Fig. 4 displayed incorrect magnification scales.

**Fig. 4:**
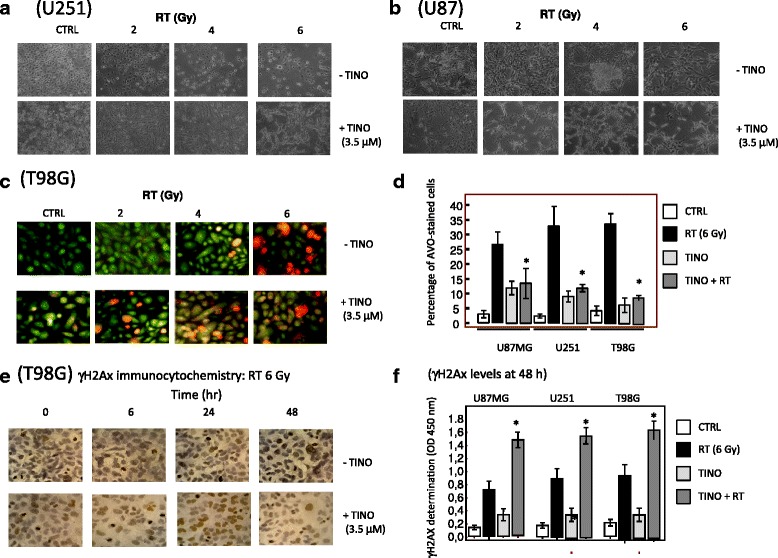
Morphological and functional effects of TINO when combined with RT in glioma cell lines. a Morphological changes observed in U251 cells after treatments with 2, 4, and 6 Gy (RT) alone or in association with TINO 3.5 μM. b Morphological changes observed in U87MG cells after treatments with 2, 4, and 6 Gy (RT) alone or in association with TINO 3.5 μM. c Representative expression of acidic vacuolar organelle (AVO) accumulation in T98G cells cultured with 2, 4, and 6 Gy (RT) alone or in association with TINO 3.5 μM. d Percentage of AVO-stained cells in U87MG, U251, and T98G cells cultured with 2, 4, and 6 Gy (RT) alone or in association with TINO 3.5 μM. Statistical analysis: *p < 0.005 in the comparison between combined TINO + RT treatment and RT alone. Single results are representative of three different experiments performed in triplicate. e Immunocytochemical evaluation of γH2Ax stain in T98G cells cultured with 4, 6, and 8 Gy (RT) alone or in association with TINO 3.5 μM. f Quantization of γH2AX expression in U87MG, U251, and T98G cells treated with RT with or without TINO. Statistical analyses: *p < 0.005 in the comparison between RT + TINO and RT or TINO treatments alone. Single results are representative of three different experiments performed in triplicate

This has now been corrected, and can be seen ahead and in the original article [1].
