# Supplementary figures and images for: The first-in-class alkylating deacetylase inhibitor molecule tinostamustine shows antitumor effects and is synergistic with radiotherapy in preclinical models of glioblastoma
Source: J Hematol Oncol. 2018 Feb 27;11:32. doi: 10.1186/s13045-018-0576-6 (PMC5830080; doi:10.1186/s13045-018-0576-6)

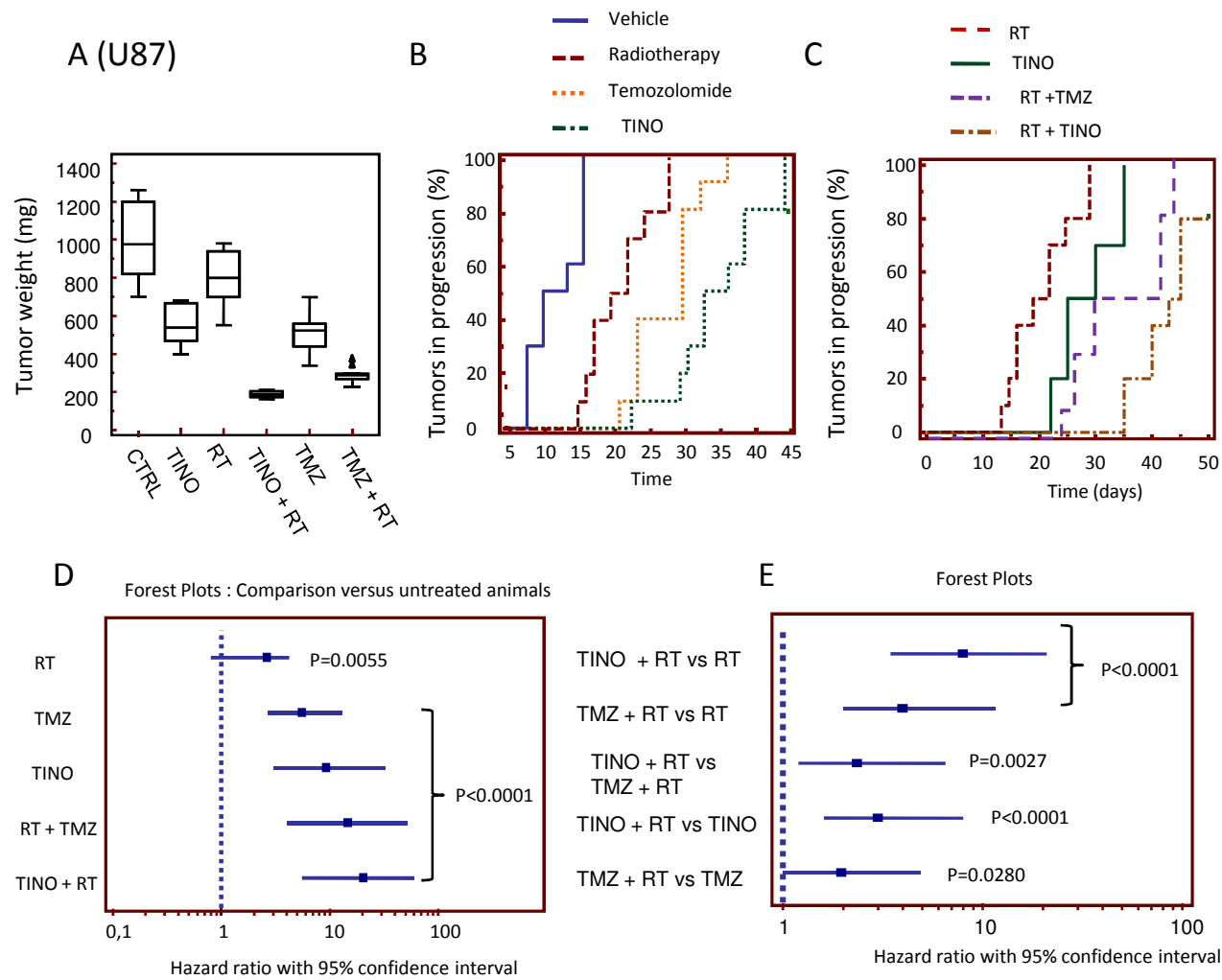

Figure S1

Supplement: Supplementary file 1 — Figure S1. Radiosensitizing effects of TINO on tumor weight and time to progression in U87MG xenograft model. (A) Analysis of tumor weights harvested at the end of experiment (50th day from randomization) in U251MG xenografts; (B) Kaplan-Meier curves generated for time to progression in U251: comparisons amongst RT, TMZ and TINO single therapies; (C) Kaplan-Meier curves generated for time to progression: analysis of radio-sensitizing effects of TINO for U251G xenografts in comparison with TMZ and RT + TMZ; (D) Forest plots (U87MG) for the comparison of TTP distribution in different treatments with untreated animals (vehicle); (E) Forest plots (U87MG) for comparison of TTP in the different combination regimens. (PDF 126 kb) [file 13045_2018_576_MOESM1_ESM.pdf]
