# Supplementary material for: The first-in-class alkylating deacetylase inhibitor molecule tinostamustine shows antitumor effects and is synergistic with radiotherapy in preclinical models of glioblastoma
Source: J Hematol Oncol. 2018 Feb 27;11:32. doi: 10.1186/s13045-018-0576-6 (PMC5830080; doi:10.1186/s13045-018-0576-6)
Supplement: Supplementary file 2 — Table S1. Additional hazard ratio analyses in xenograt models. Table S2 Additional hazard ratio analyses in orthotopic U251models. Table S3 Additional hazard ratio analyses in orthotopic CSCs-5model. (DOCX 23 kb) [file 13045_2018_576_MOESM2_ESM.docx]

**Table S1. Additional hazard ratio analyses in xenograt models.**

| Comparison | U87MG | | | U251 | | | T98G | |  |
| --- | --- | --- | --- | --- | --- | --- | --- | --- | --- |
|  | Hazard Ratio | 95% CI | Significance | Hazard ratio | 95% CI | Significance | Hazard Ratio | 95% CI | Significance |
| CTRL vs RT | 7.2 | 2.4 to16.7 | P<0.0001 | 5.4 | 1.1 to 8.7 | P<0.0001 | 2.8 | 1.0 to 7.5 | P=0.0043 |
| CTRL vs EDO | 20.2 | 6.7 to 44.8 | P<0.0001 | 16.8 | 7.0 to 45.6 | P<0.0001 | 7.8 | 2.3 to 15.5 | P<0.0001 |
| CTRL vs TMZ | 14.6 | 4.2 to 24.5 | P<0.0001 | 9.9 | 3.3 to 19.5 | P<0.0001 | 2.1 | 0.8 to 6.4 | P=0.3720 NS |
| RT vs EDO | 16.4 | 4.5 to 28.5 | P<0.0001 | 9.4 | 2.5 to 15.8 | P<0.0001 | 4.3 | 1.6 to 9.5 | P<0.0001 |
| RT vs TMZ | 3.3 | 1.5 to 13.0 | P<0.0001 | 6.3 | 2.1 to 12.1 | P<0.0001 | 1.9 | 0.8 to 4.8 | P=0.0628 NS |
| CTRL vs EDO+RT | 29.2 | 7.5 to 84.2 | P<0.0001 | 28.2 | 6.5 to 48.5 | P<0.0001 | 9.6 | 2.5 to 16.6 | P<0.0001 |
| CTRL vs TMZ+RT | 22.0 | 2.1 to 61.0 | P<0.001 | 15.5 | 4.7 to 27.8 | P<0.0001 | 4.9 | 1.3 to 9.7 | P<0.0001 |
| RT vs TMZ + RT | 3.7 | 1.3 to 10.9 | P=0.0030 | 22.0 | 6.6 to 41.8 | P<0.0001 | 3.6 | 1.2 to 9.7 | P=0.0015 |
| TMZ vs TMZ + RT | 1.7 | 1.2 to 3.3 | P=0.0863 NS | 7.9 | 2.5 to 14.2 | P<0.0001 | 2.5 | 1.0 to 6.7 | P=0.0097 |

**Table S2. Additional hazard ratio analyses in orthotopic U251models.**

| Comparison | U251 | | | | | |
| --- | --- | --- | --- | --- | --- | --- |
|  | **Disease free survival** | | | **Overall survival** | | |
|  | Hazard ratio | 95% CI | Significance | Hazard ratio | 95% CI | Significance |
| CTRL vs RT | 3.5 | 1.3 to 15.0 | P=0.0029 | 2.8 | 1.0 to 7.7 | P=0.0241 |
| CTRL vs EDO | 8.0 | 2.2 to 18.5 | P<0.0001 | 6.6 | 1.8 to 14.7 | P<0.0001 |
| CTRL vs TMZ | 6.5 | 1.9 to 13.2 | P<0.0001 | 5.8 | 2.8 to 12.7 | P<0.0001 |
| RT vs EDO | 7,.5 | 2.6 to 17.9 | P<0.0001 | 5.2 | 1.2 to 9.6 | P<0.001 |
| RT vs TMZ | 7.0 | 2.0 to 14.7 | P=0.0123 | 3.8 | 1.6 to13.4 | P=0.0004 |
| CTRL vs EDO+RT | 10.0 | 3.7 to 38.5 | P<0.0001 | 8.9 | 2.0 to 18.3 | P<0.0001 |
| CTRL vs TMZ+RT | 9.0 | 1.7 to 31.8 | P<0.0001 | 7.3 | 2.5 to 17.9 | P<0.0001 |
| RT vs TMZ + RT | 7.6 | 1.2 to 18.5 | P=0.0023 | 4.8 | 1.6 to 9.0 | P<0.0001 |
| TMZ vs TMZ + RT | 2.6 | 1.0 to 8.2 | P=0.0188 | 2.9 | 1.2 to 7.1 | P=0.0179 |

**Table S3. Additional hazard ratio analyses in orthotopic CSCs-5model.**

| Comparison | U251 | | | | | |
| --- | --- | --- | --- | --- | --- | --- |
|  | **Disease free survival** | | | **Overall survival** | | |
|  | Hazard ratio | 95% CI | Significance | Hazard ratio | 95% CI | Significance |
| CTRL vs RT | 2.2 | 1.3 to 8.0 | P=0.0258 | 1.8 | 0.8 to 4.7 | P=0.0835 |
| CTRL vs EDO | 6.0 | 1.6 to 17.2 | P<0.0001 | 5.8 | 1.3 to 16.2 | P<0.0001 |
| CTRL vs TMZ | 4.8 | 1.4 to 13.8 | P<0.0001 | 5.0 | 2.5 to 15.7 | P<0.0001 |
| RT vs EDO | 4.2 | 1,2 to 12.6 | P<0.0001 | 4.8 | 1.6 to 17.3 | P<0.0001 |
| RT vs TMZ | 3.5 | 1.2 to 10.2 | P=0.0030 | 3.5 | 1.6 to 14.4 | P=0.0021 |
| CTRL vs EDO+RT | 8.8 | 2.3 to 26.0 | P<0.0001 | 10.5 | 3.6 to 36.5 | P<0.0001 |
| CTRL vs TMZ+RT | 7.2 | 1.7 to 19.4 | P<0.0001 | 9.0 | 2.5 to 17.9 | P<0.0001 |
| RT vs TMZ + RT | 5.2 | 1.6 to 16.55 | P<0.0001 | 5.6 | 1.1 to 9.0 | P<0.0001 |
| TMZ vs TMZ + RT | 3.7 | 1. to 10.2 | P<0.0001 | 3.9 | 1.2 to 12.1 | P=0.0179 |
